# Genetic basis of negative heterosis for growth traits in chickens revealed by genome-wide gene expression pattern analysis

**DOI:** 10.1186/s40104-021-00574-2

**Published:** 2021-04-18

**Authors:** Chunning Mai, Chaoliang Wen, Zhiyuan Xu, Guiyun Xu, Sirui Chen, Jiangxia Zheng, Congjiao Sun, Ning Yang

**Affiliations:** National Engineering Laboratory for Animal Breeding and Key Laboratory of Animal Genetics, Breeding and Reproduction, Ministry of Agriculture and Rural Affairs, China Agricultural University, Beijing, 100193 China

**Keywords:** Chicken, Growth, Gene expression patterns, Heterosis, Oxidative phosphorylation

## Abstract

**Background:**

Heterosis is an important biological phenomenon that has been extensively utilized in agricultural breeding. However, negative heterosis is also pervasively observed in nature, which can cause unfavorable impacts on production performance. Compared with systematic studies of positive heterosis, the phenomenon of negative heterosis has been largely ignored in genetic studies and breeding programs, and the genetic mechanism of this phenomenon has not been thoroughly elucidated to date. Here, we used chickens, the most common agricultural animals worldwide, to determine the genetic and molecular mechanisms of negative heterosis.

**Results:**

We performed reciprocal crossing experiments with two distinct chicken lines and found that the body weight presented widely negative heterosis in the early growth of chickens. Negative heterosis of carcass traits was more common than positive heterosis, especially breast muscle mass, which was over − 40% in reciprocal progenies. Genome-wide gene expression pattern analyses of breast muscle tissues revealed that nonadditivity, including dominance and overdominace, was the major gene inheritance pattern. Nonadditive genes, including a substantial number of genes encoding ATPase and NADH dehydrogenase, accounted for more than 68% of differentially expressed genes in reciprocal crosses (4257 of 5587 and 3617 of 5243, respectively). Moreover, nonadditive genes were significantly associated with the biological process of oxidative phosphorylation, which is the major metabolic pathway for energy release and animal growth and development. The detection of ATP content and ATPase activity for purebred and crossbred progenies further confirmed that chickens with lower muscle yield had lower ATP concentrations but higher hydrolysis activity, which supported the important role of oxidative phosphorylation in negative heterosis for growth traits in chickens.

**Conclusions:**

These findings revealed that nonadditive genes and their related oxidative phosphorylation were the major genetic and molecular factors in the negative heterosis of growth in chickens, which would be beneficial to future breeding strategies.

**Supplementary Information:**

The online version contains supplementary material available at 10.1186/s40104-021-00574-2.

## Background

Heterosis, first proposed by Shull in 1908 [1], is defined as the deviation between F_1_ reciprocal crosses and their parental lines mean [2]. Heterosis has become a routine strategy for livestock and crop breeding and has driven great improvements in performance or livability over the last century. The fundamental mechanism underlying heterosis will determine whether it can be manipulated for the benefit of agriculture and biotechnology. Numerous studies have attempted to explain the genetic mechanism of heterosis, and three classic quantitative genetic hypotheses have been proposed: dominance [3, 4], overdominance [1, 5], and epistasis [6, 7]. However, these three models were mainly theoretical and could not provide a full explanation for the molecular basis and physiological causes of heterosis [8, 9].

At the molecular level, variation in gene expression is thought to constitute a significant source of phenotypic diversity [10]. Investigation of differentially expressed genes (DEGs) between crossbred and their parent lines might contribute to improving our understanding of the molecular basis for heterosis. In particular, the gene expression patterns involved in some metabolic pathways are obviously correlated with heterotic phenotypes. For example, several studies on heterosis in rice revealed that differentially expressed genes between hybrids and their parents were involved in energy metabolism, which contributed in a significant way to the increased yield of hybrids [11, 12]. Fujimoto et al. [13] demonstrated that the higher photosynthetic efficiency of *Arabidopsis* hybrids was obviously associated with the early increase in the activity of genes involved in chlorophyll biosynthesis and photosynthesis, which contributed to increased heterotic biomass. Similar studies were also reported in animals. Hedgecock et al. [14] conducted transcriptomic analysis in *Crassostrea gigas* and found that nonadditive genes and their related protein metabolism played important roles in growth heterosis.

In addition to positive heterosis, negative heterosis is also observed in many farm animals [15–19] and plants [20–23]. Negative heterosis can cause unfavorable impacts on production performance in agriculture, such as negative heterosis of body weight in quails [15, 16], carcass traits in beefs [17], hybrid necrosis in plants [20, 21], and hybrid weakness of shoot dry weight [22] and height [23] in rice. The poultry industry has a long history of using crosses between different populations to take advantage of strain complementarity. In theory, the magnitude of heterosis is inversely correlated to the extent of genetic similarity between parental lines, and interspecific crosses show greater heterosis than intraspecific crosses [24]. However, abundant evidence has revealed the existence of negative heterosis for growth traits in chickens when the genetic background of the parents varies greatly. Williams et al. [25] used the high- and low-body-weight chicken lines from Virginia Tech for heterosis analysis, and approximately − 24% and − 16% heterosis was observed for the body weights of reciprocal crosses at 4 and 8 weeks of age, respectively. Similar results were reported by Jull and Quinn [26], Maw [27], Liu et al. [28], and Sutherland et al. [29]. Negative heterosis for carcass performance, especially for muscle mass in reciprocal crosses, was reported by Sun et al. [30] when broiler and layer chickens were used as parents.

However, the genetic and molecular basis of heterosis for growth traits in chickens is still a mystery. Chickens are the most common and widespread domestic animals worldwide, as well as a great source of meat for humans. Revealing the genetic mechanisms of negative heterosis for growth traits will improve yield to meet the chicken meat demands of humans worldwide. Breast muscle is the largest proportion of body weight and is an important indicator of the growth rate in chickens [31]; thus, investigating the negative heterosis of breast muscle mass can be a breakthrough to explore this unclear phenomenon. The development of transcriptome sequencing technologies has allowed unbiased and reproducible sequencing of whole transcriptomes, which are valuable for characterizing the patterns of gene expression and have been used to unravel the mechanisms of heterosis [32–34]. In the current study, Cornish (C) and Rhode Island White (R) were used as parental lines to produce the F_1_ generation. Cornish, as a standard broiler breed, has been selected for growth and, in particular, for muscle growth. Rhode Island White, a layer breed, has been intensively selected for egg production. The RNA-sequencing strategy was used to identify the transcriptomic differences in the breast muscle of reciprocal crosses and their parental lines. The objective of this study was to provide new insight into the molecular basis of negative heterosis for growth performance in chickens.

## Methods

### Experimental populations

**Fig. 1:**
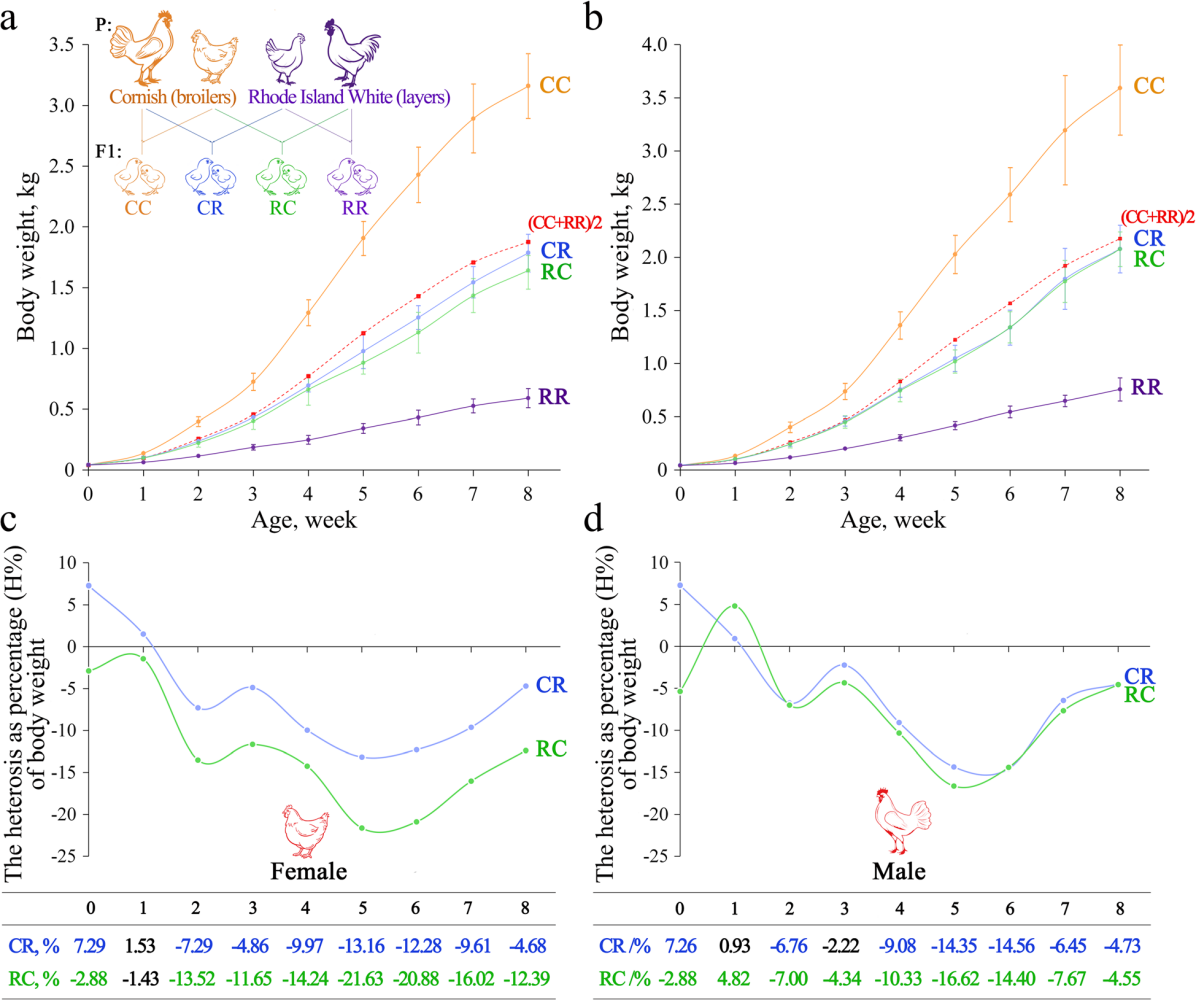
Body weight for purebred and crossbred progenies and heterosis of body weight for reciprocal crosses. A schematic diagram of two pure lines, Rhode Island White (R, layers) and Cornish (C, broilers), used as parental lines to produce the F1 generation (CC, CR, RC and RR), is shown in the upper left corner of the figure. Body weight of females (**a**) and males (**b**) from hatching to 8 weeks of age. Heterosis as a percentage (H%) of body weight for females (**c**) and males (**d**) in reciprocal crosses. The values of H% of body weight are shown below the figure. Values presented in blue and green indicate that H% was highly significant (*P* < 0.01). Values presented in black indicate that H% was not statistically significant (*P* > 0.05)

Two domesticated chicken breeds, Cornish (meat-type chicken, C line) and Rhode Island White (egg-type chicken, R line), from Beijing Huadu Yukou Poultry Industry Co., Ltd. were employed as parents in this study to produce purebred progenies and reciprocal crosses (Fig. 1a). The C line has been selected for 42-day body weight for 7 generations, whereas the R line has been intensively selected for total egg production to 300 days of age for 15 generations. We selected 10 males and 120 females from the 8th generation of the C line and 10 males and 80 females from the 16th generation of the R line as parents according to the following criteria: (i) cocks in each line with similar body weight and good semen quality and (ii) hens in each line with similar body weight and high egg production. These chickens were housed with individual cages in the same poultry facility. Each male (both C and R) was mated with 6 C and 4 R females by artificial insemination. The eggs were collected and recorded daily. Finally, a total of 632 chicks (347 for females and 285 for males) with clear pedigree information were hatched on the same day and used for subsequent studies.

### Phenotypic measurement and sample collection

At hatching, chicks were identified as males and females by vent sexing and then reared in separated cages under the same environment with free access to feed and water. The hatched chicks were wing-banded for individual identification. The four genetic combinations were reared in different cages to eliminate size disparities and reduce competition. The body weights were measured weekly from hatch to 8 weeks of age (Table S1). The length of the left shank and sternum for chickens were measured at 3, 6, and 8 weeks of age (Table S2). At 6 weeks of age, we randomly selected 64 female chickens (14, 14, 16 and 20 for CC, RR, CR and RC, respectively) and 44 male chickens (10, 9, 15 and 10 for CC, RR, CR and RC, respectively) from different half-sib families. These chickens were euthanized by cervical dislocation, and the following carcass traits were measured by an electronic balance: slaughter weight (measured after bloodletting), both left and right wing weight, breast muscle weight (pectoralis major and minor), drumstick weight (bone and muscle), and drumstick bone weight. The drumstick muscle weight was calculated as the drumstick weight subtracted from the drumstick bone weight. Heterosis as a percentage (H%) of the abovementioned traits was calculated according to the following equation:

A $$ H\%=\frac{\overline{F_1}-\left(\overline{P_M}+\overline{P_F}\right)/2}{\left(\overline{P_M}+\overline{P_F}\right)/2}\times 100\% $$

where $\overline{F_1}$, $\overline{P_M}$ and $\overline{P_F}$ are the mean phenotypes of the reciprocal crosses, the maternal and paternal lines, respectively. In order to evaluate the significance of H%, Student’s t-value was estimated based on the formula of Wu et al. [35]:

B $$ \mathrm{t}=\frac{H\%}{2\sqrt{\frac{\sum {\left({F}_{1i}-\overline{F_1}\right)}^2}{N-1}}/\left[\left(\overline{P_M}+\overline{P_F}\right)\times \sqrt{N}\ \right]} $$

where *F*_1*i*_ is the phenotype of individual *i* from reciprocal crosses; *N* is the number of birds in RC or CR. We obtained the *P*-value using the pt. function in the R program (https://www.r-project.org/) according to the *t*-value and the degrees of freedom. H% was considered significant and highly significant if *P*-value < 0.05, and *P*-value < 0.01, respectively. Meanwhile, six female offspring (from 4 –6 half-sib families) of each group, except for five female offspring in the RC group, were selected, and the left pectoralis major muscle of these chickens was isolated for subsequent RNA sequencing.

### RNA extraction and sequencing

Total RNA was extracted using the TRIzol® Reagent (Invitrogen, USA) according to the manufacturer’s instructions and then dissolved in DEPC-treated water. To ensure that RNA was isolated successfully, the extracted RNA was first evaluated by 1% agarose gel electrophoresis. Then, the RNA purity, concentration and integrity of all eligible RNA extraction were determined by a NanoPhotometer® spectrophotometer (IMPLEN, CA, USA), a Qubit®2.0 Fluorimeter (Life Technologies, CA, USA), and an RNA Nano 6000 Assay Kit from the Bioanalyzer 2100 system (Agilent Technologies, CA, USA), respectively. The samples with an RNA integrity number value that greater than 7.0 were considered as high-quality RNA samples. A total of 23 samples were qualified for RNA sequencing library construction. Approximately 3 μg of RNA per sample was subjected to RNA-seq library construction using the NEBNext® UltraTM RNA Library Prep Kit (Illumina, USA) according to the manufacturer’s guide. After PCR amplification and purification, 150 bp paired-end sequencing was performed on the Illumina Hiseq X Ten platform (Illumina Inc., San Diego, CA, USA) and generated in nearly 750.13 million raw reads.

### Quality control and mapping

To minimize mapping errors, reads that met the following parameter were removed: a) containing adaptors; b) with more than 10% unknown nucleotides; c) with more than 50% low-quality bases (Qphred ≤20). The chicken reference genome (galGal5) and gene model annotation files were downloaded from the Ensembl database (ftp://ftp.ensembl.org/pub/release-91/). After quality control, over 721.42 million high quality reads with Q20 > 95% (Table S3) were aligned to the chicken reference genome using Hisat2 (v2.0.5) [36]. Approximately 76% of the high quality reads in each sample were mapped to the reference genome. Over 80% of reads were assigned to exonic regions, approximately 4% were assigned to intronic regions, and 16% were assigned to intergenic regions.

### Differential gene expression analyses

The mapped reads of each sample were assembled by StringTie (v.1.3.3b) [37]. The function of novel genes was annotated based on the Pfam database (v.31.0) [38]. Then, the gene count matrix table was generated by featureCounts (v1.5.0-p3) [39]. FPKM (fragments per kilobase million) values were extracted from the StringTie outputs. To enhance the statistical power for DEGs, the genes with an average FPKM < 1 were removed. Meanwhile, the sex-linked genes were removed from the following analysis. After these steps, 11,050 genes were filtered, and the remaining 11,544 genes were used for differential expression analysis between two purebred lines (CC vs. RR) and between reciprocal crosses and purebred lines (CR vs. CC, CR vs. RR, RC vs. CC and RC vs. RR) using the DESeq2 package (v.1.16.1) [40] in R project. We presented DESeq2, a method for differential analysis of count data, using the empirical Bayes shrinkage method to estimate dispersions and fold changes. The *P*-value was calculated by the Wald test. To control the false discovery rate, the resulting *P*-values were adjusted for multiple testing using the Benjamini-Hochberg method. Genes with an adjusted *P*-value < 0.05 were considered differentially expressed genes in the corresponding comparison.

### Evaluation of differential inheritance patterns

We used the average FPKM value of each group and the adjusted *P*-value to evaluate different inheritance patterns of genes (Table S4) [32]. These genes were further classified into three inheritance patterns: additivity, dominance and overdominance, based on the level of gene expression exhibited by reciprocal crosses and parental lines. In brief, additivity (I and XII) occurred when the gene expression was significantly different between the two parental lines (adjusted *P*-value < 0.05), and the gene expression of reciprocal crosses (CR or RC) was higher than one parental line but lower than the other parental line. Gene expression within CR/RC that was not significantly different from one parental line but significantly higher (or lower) than the other parental line was regarded as dominance (II, IV, IX, and XI). Gene expression within CR/RC that was significantly higher (or lower) than both parental lines (CC and RR) was considered overdominance (V, VI, VIII, III, VII, and X).

To confirm the reliable of gene expression patterns reveled by RNA-seq, we performed quantitative real-time PCR (qRT-PCR) experiments. qRT-PCR reactions were performed with three technical replicates for each individual. The details of qRT-PCR and related results have been previously described [34].

### GO enrichment and KEGG pathway analyses

To investigate the biological function of nonadditive genes involved, we performed functional enrichment analysis, including Gene Ontology (GO) categories and Kyoto Encyclopedia of Genes and Genomes (KEGG) pathways, using the Clusterprofile package [41] in the R project. The GO terms and KEGG pathways with FDR < 0.05 (BH method) were considered significant.

### ATP content assay

ATP content was detected using an ATP assay kit (S0026B, Beyotime Biotechnology, China) as described in a previous study [42]. The method is based on the theory that luciferase catalyzes luciferin to form fluorescence, which requires energy provided by ATP. Thus, the emitted fluorescence intensity is linearly related to the ATP concentration. Briefly, tissue samples (20 mg) were homogenized on ice with 150 μL of ice-cold assay buffer. It was then centrifuged at 12,000 r/min for 10 min at 4 °C to remove insoluble materials, and the supernatant was collected. An aliquot (100 μL) of ATP detection working solution was added to each well of a white 96-well plate. After incubation for 3 min at room temperature, 50 μL of supernatant was added to the wells. Luminescence was measured by a fluorescence microplate reader.

### ATPase activity assay

ATPase activity was assessed using an ATPase activity assay kit (MAK113, Millipore Sigma, St. Louis, MO, USA) according to the manufacturer’s instructions. ATPase hydrolyzes ATP into ADP and free phosphate. Free phosphate causes the malachite green reagent to form a stable dark green colorimetric product that is proportional to the ATPase activity. In brief, tissue samples (20 mg) were homogenized on ice with 200 μL of ice-cold assay buffer. They were then centrifuged at 14,000 r/min for 10 min at 4 °C to remove insoluble materials, and the supernatant was collected. An aliquot (30 μL) of the reaction mixture solution was added to each well of a 96-well flat-bottom plate and incubated for 30 min at room temperature. Then, 200 μL of reagent was added to each well and incubated for an additional 30 min at room temperature to terminate the enzyme reaction. Finally, the absorbance was determined at 620 nm for all samples.

### Statistical analysis

Differences in breast muscle weight, ATP content and ATPase activity among parental lines and reciprocal crosses were assessed using ANOVA followed by Tukey’s HSD test in the R program. The results were considered to be statistically significant when the adjusted *P*-value was less than 0.05.

## Results

### Negative Heterosis of body weights and carcass traits

As described in Fig. 1a, we chose the C and R breeds to produce purebred (CC and RR) and reciprocal crossbred progenies (RC and CR). The body weight of each progeny was measured weekly from hatching to 8 weeks of age. The traits of shank length and sternum length were measured at 3, 6, and 8 weeks of age. The correlation among body weights at different ages for females and males varied from 0.115 to 0.991 and from 0.012 to 0.991, respectively (Fig. S1). As shown in the dynamic growth of parental lines and reciprocal crosses, the body weights of females and males in CR and RC from 2 to 8 weeks of age were lower than the average of CC and RR (Fig. 1a, b and Table S1), although reciprocal crosses exhibited slightly positive heterosis for the length of shank and sternum at 6 and 8 weeks of age (Table S5). The degree of heterosis for body weight is displayed in Fig. 1c and d in terms of heterosis as a percentage. The H% of body weight varied from − 21.63% to 7.29% and from − 16.62% to 7.26% for females and males, respectively. The negative heterosis of females and males reached a maximum value between the fifth and sixth weeks of age. In females, compared with CR (range of − 13.16% to 7.29%), the H% was smaller in RC, which varied from − 21.63% to − 1.43%. In males, compared with CR (range of − 14.56% to 7.26%), the H% was smaller in RC, which varied from − 16.62% to 4.82%. In CR (females) and RC (males), the H% of body weight decreased from hatching to 5 weeks of age and showed a slight increasing trend from 5 to 8 weeks of age, while in RC (females) and CR (males), the inflection point was 6 weeks of age.

**Fig. 2:**
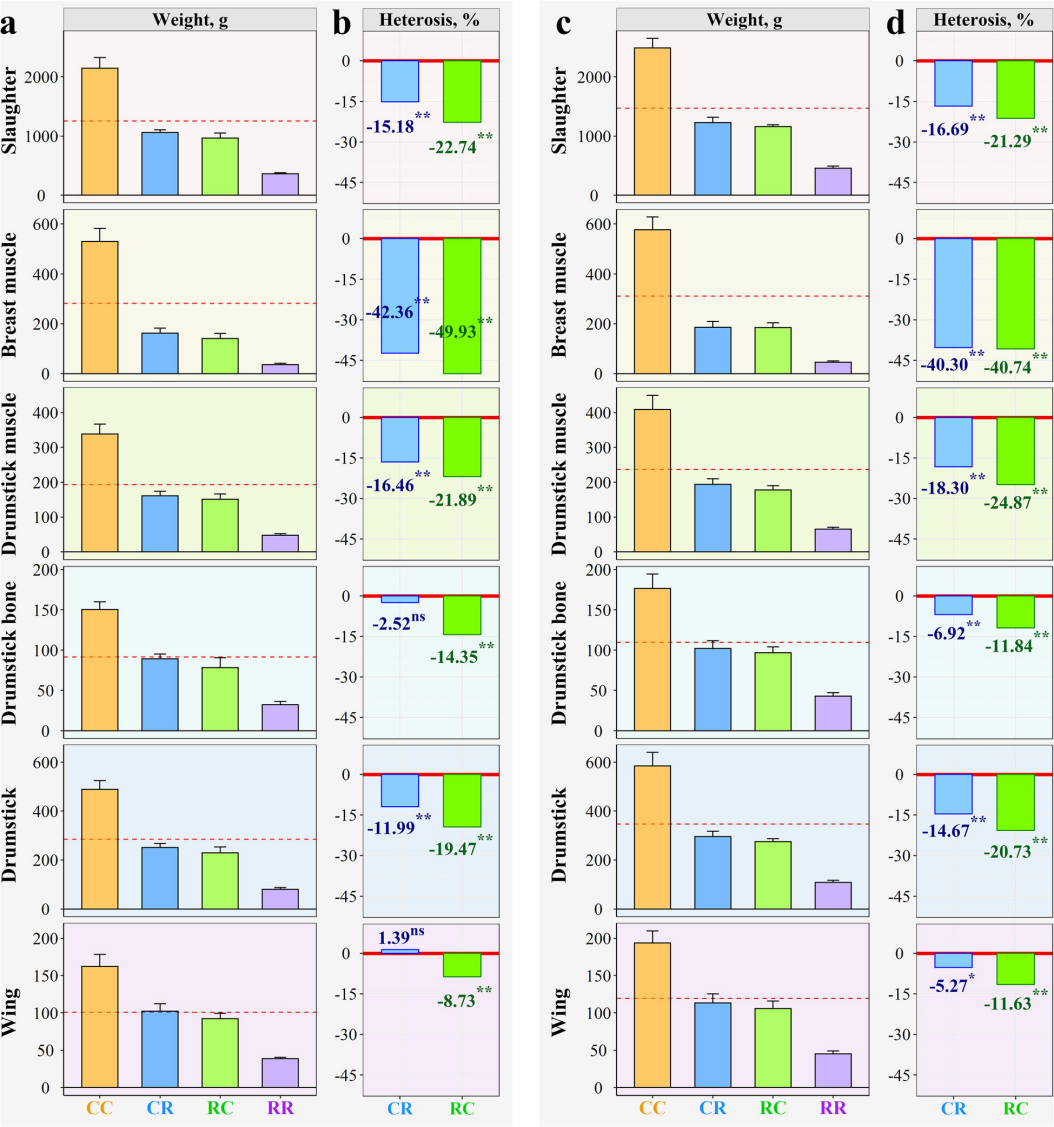
Heterosis of carcass performance for purebred and crossbred progenies at 6 weeks of age. The breast muscle weight, drumstick bone weight, drumstick weight (bone and muscle) and wing weight were measured on both sides. The drumstick muscle weight was calculated as the drumstick weight subtracted from the drumstick bone weight. **a-b** Female. **c**-**d** Male. For (**a**) and (**c**), the dashed red line represents the mid-parent value. For (**b**) and (**d**), ns, * and ** indicate that the heterosis as a percentage (H%) was not statistically significant (*P* > 0.05), significant (*P* < 0.05) and highly significant (*P* < 0.01), respectively

Given that fast growing broilers are mostly marketed at 6 weeks of age, we randomly slaughtered 108 chickens from four groups for carcass composition analysis at 42 days of age. As shown in Fig. 2 and Table S6, most carcass traits, including slaughter weight, breast muscle weight, drumstick weight, and drumstick muscle weight, showed extremely significant negative heterosis (*P* < 0.01) in reciprocal crosses of females and males. Among these various carcass traits, the negative heterosis of breast muscle weight was the largest, e.g., − 42.35% (CR) and − 49.93% (RC) in females and − 40.29% (CR) and − 40.75% (RC) in males. Meanwhile, the correlation of body weight and breast muscle weight at 6 weeks of age was 0.98 for both females and males (Fig. S2).

### Inheritance of gene expression in reciprocal crosses

**Fig. 3:**
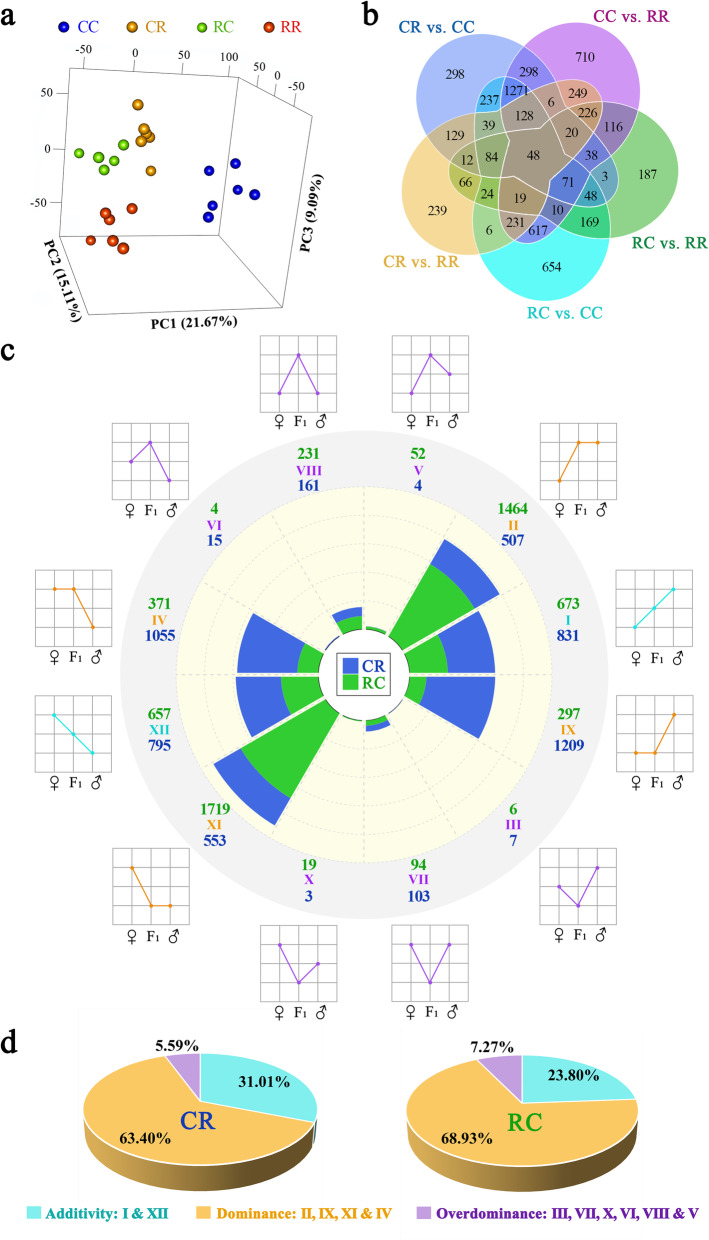
Analysis of gene inheritance patterns. **a** Principal component analysis of the reciprocal crosses (CR, RC) and the parental lines (RR, CC). **b** The number of DEGs among F_1_ progenies. **c** Inheritance patterns of DEGs between reciprocal crosses and parental lines. DEGs were divided into 12 types, e.g., class I, II, III, IV, V, VI, VII, VIII, IX, X, XI and XII, and further classified into three inheritance patterns: additivity (class I and XII), dominance (class II, IV, IX, and XI) and overdominance (class III, V, VI, VII, VIII, and X), based on the level of gene expression exhibited by reciprocal crosses and parental lines. Additivity, dominance, and overdominance are presented in blue, orange, and purple, respectively. Each class was accompanied by diagrams representing the relative expression levels of the maternal line (left dot), F_1_ (middle dot), and paternal line (right dot). The number of DEGs in each class is shown above this class (green numbers, represented as the RC group) and below (blue numbers, represented as the CR group). **d** The proportion of additive, dominant and overdominant genes in DEGs

As noted above, the negative heterosis of body weight and carcass traits were widespread in the present study. The more fundamental question is why the reciprocal progenies exhibited this phenomenon. Thus, the transcriptional data of breast muscle tissues for the four groups were used to analyze the differences in gene expression between parental lines and reciprocal crosses. A principal component analysis (PCA) was performed to visualize the differences in gene expression. The PCA plot showed that the four groups were obviously separated from each other (Fig. 3a), indicating that there were visible differences in gene expression between the two parental lines and between the parental lines and reciprocal crosses.

A total of 6253 DEGs between the two parental lines and between the reciprocal crosses and parental lines were identified (Fig. 3b and Fig. S3), e.g., 5147 (RR vs. CC), 3205 (CR vs. CC), 1628 (CR vs. RR), 1184 (RC vs. RR) and 4470 (RC vs. CC). These DEGs were divided into 12 types (I, II, III, IV, V, VI, VII, VIII, IX, X, XI and XII; for details, see Table S4) based on the level of gene expression exhibited by reciprocal crosses and parental lines. The number of the 12 type genes in the CR and RC groups is shown in Fig. 3c and Table S7. The 12 types were further classified into 3 main inheritance patterns: additivity (I, XII), dominance (II, IV, IX and XI), and overdominance (III, V, VI, VII, VIII and X). The number of dominant genes was 3324 and 3851 in CR and RC, respectively. The number of overdominant genes was 293 and 406 in RC and CR, respectively. Nonadditivity, including dominance and overdominace, was the major gene inheritance pattern. Nonadditive genes accounted for 68.99% and 76.20% of DEGs in CR and RC, respectively (Fig. 3d).

### Nonadditive inheritance is related to oxidative phosphorylation

**Fig. 4:**
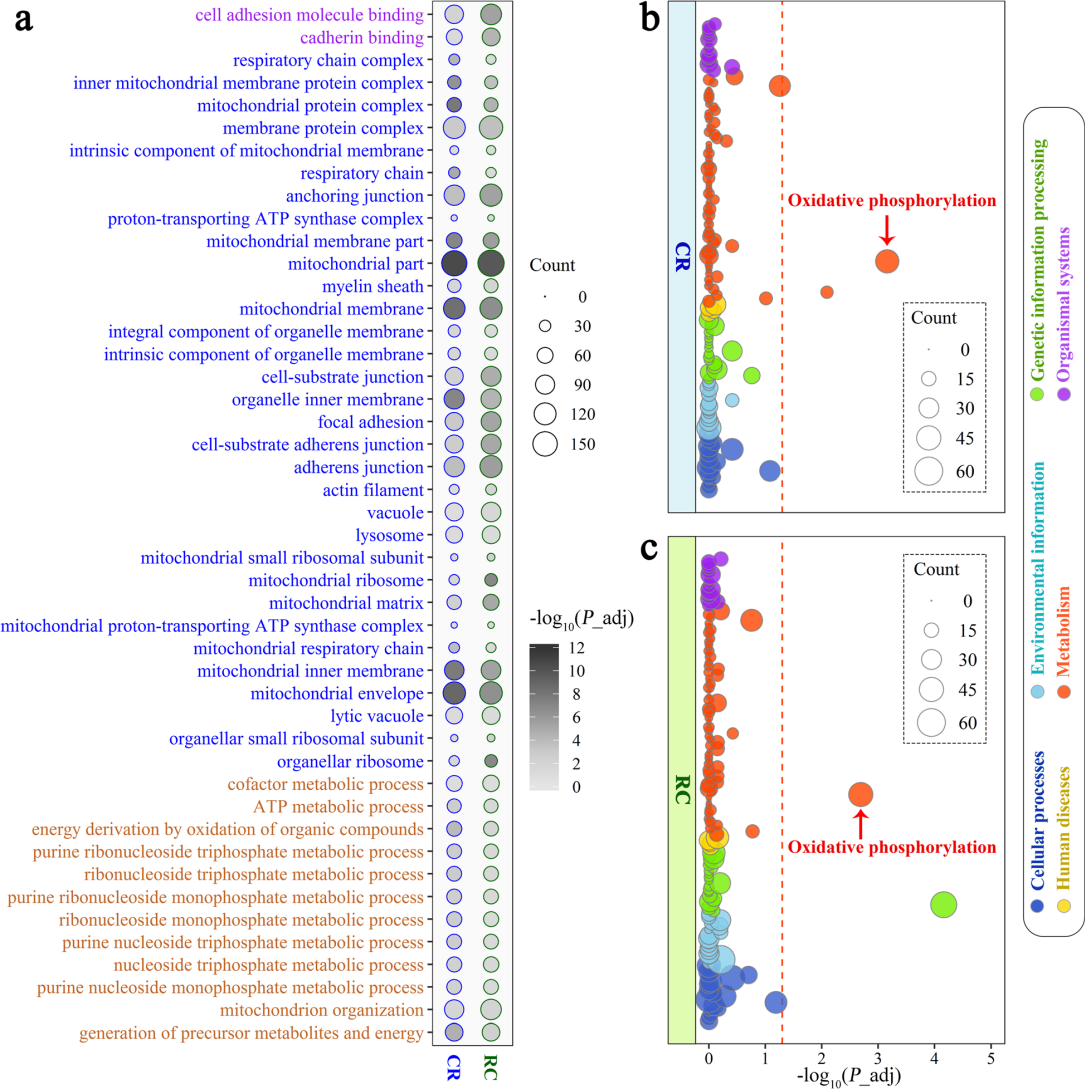
Functional enrichment analysis for dominant genes. **a** Significant GO terms of dominant genes in reciprocal crosses. Each dot represents a GO term, and the size of a dot represents the number of genes enriched in the GO terms. The shade of the colored dots indicates the level of significance of the GO terms. The names of GO terms in purple, blue and brown represent the GO terms that belonged to molecular function, cell composition and biological process, respectively. **b** and **c** KEGG pathway analysis for dominant genes in the CR and RC groups, respectively. Each dot represents a KEGG pathway, and the size of a dot represents the number of genes enriched in the pathway. The color of a dot represents the KEGG classification in the pathway. The dashed red lines indicate significance levels (adjusted *P*-value < 0.05). The dots that passed dashed red lines are regarded as significant pathways

Apart from the focus on gene expression patterns in reciprocal crosses, we are more interested in the biological processes that additive and nonadditive genes are related to. We tested for enrichment of these genes against GO and KEGG pathways to detect the metabolic pathways involved. The functional enrichment analyses showed no significant GO terms or pathways detected in additive genes of the RC group, although one KEGG pathway, ribosome, was significantly enriched in additive genes of the CR group. However, as shown in Fig. 4a, the dominant genes of CR and RC were both significantly enriched in 46 GO terms, including 2 GO terms of molecular function, 32 GO terms of cell composition and 12 GO terms of biological process. The majority of these GO terms were associated with mitochondrial components and energy metabolism. Furthermore, the KEGG pathway analysis showed that one shared pathway, oxidative phosphorylation, was significantly enriched in the dominant genes of the CR and RC groups (Fig. 4b). Additionally, overdominant genes of both RC and CR were also enriched in GO terms related to mitochondrial components and energy metabolism (Table S8). The oxidative phosphorylation pathway was also detected in the overdominant genes of the CR and RC groups (Fig. S4).

**Fig. 5:**
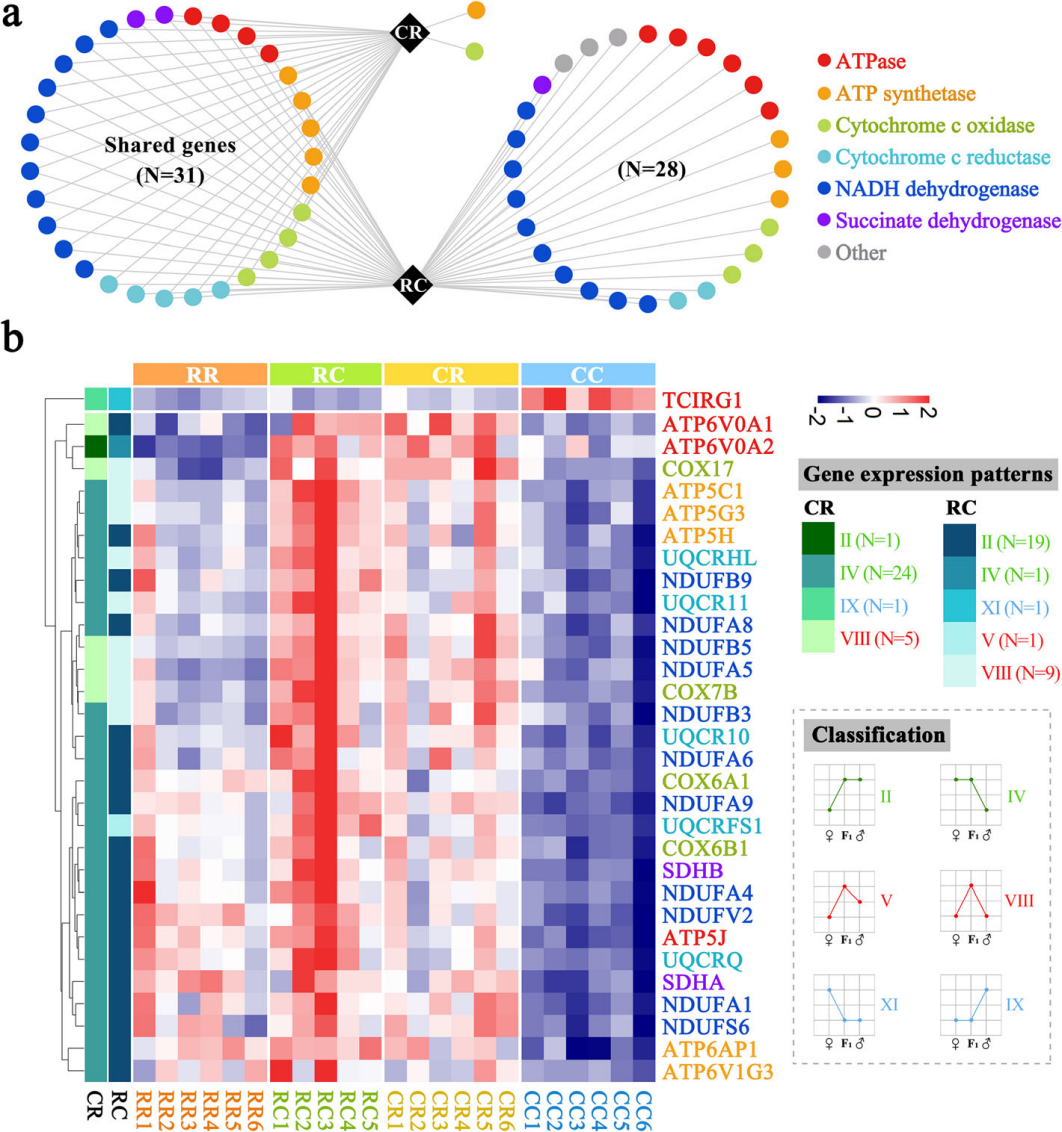
Analyses of nonadditive genes enriched in oxidative phosphorylation of reciprocal crosses. **a** Overlap of nonadditive genes enriched in oxidative phosphorylation in the CR and RC groups. Each dot represents one gene. The names of the enzymes encoded by nonadditive genes are listed on the right. **b** Heatmap of shared gene expression levels in reciprocal crosses and their parents. The types of gene expression patterns in the CR and RC are shown on the left side. Schematic diagrams of the expression patterns and the number of genes are shown on the right. The color of the gene names represents the encoded enzyme, which is the same as the enzyme in plot (**a**)

Given that nonadditive genes were significantly enriched in the pathway of oxidative phosphorylation, we further analyzed the nonadditive genes in the CR and RC groups. The number of nonadditive genes enriched in oxidative phosphorylation was 33 and 59 in the CR and RC groups, respectively (Fig. 5a). These genes were related to NADH dehydrogenase, cytochrome c reductase, cytochrome c oxidase, ATP synthase, ATPase and succinate dehydrogenase. Among those, 31 shared genes were detected in the CR and RC groups (Fig. 5a and Table S9). Considering that nonadditive genes contained 10 different types, we further analyzed which type was important to the process of oxidative phosphorylation and found that types IV and II were the major gene expression patterns in CR and RC, respectively (Fig. 5b). Type IV in CR and type II in RC accounted for 77.42% (24 of 31) and 61.29% (19 of 31) of shared genes, respectively. It is worth noting that the expression level of these genes in reciprocal progenies biased to the R line, and the expression level in the R line was significantly higher than that in the C line.

### ATP content and ATPase activity detection

**Fig. 6:**
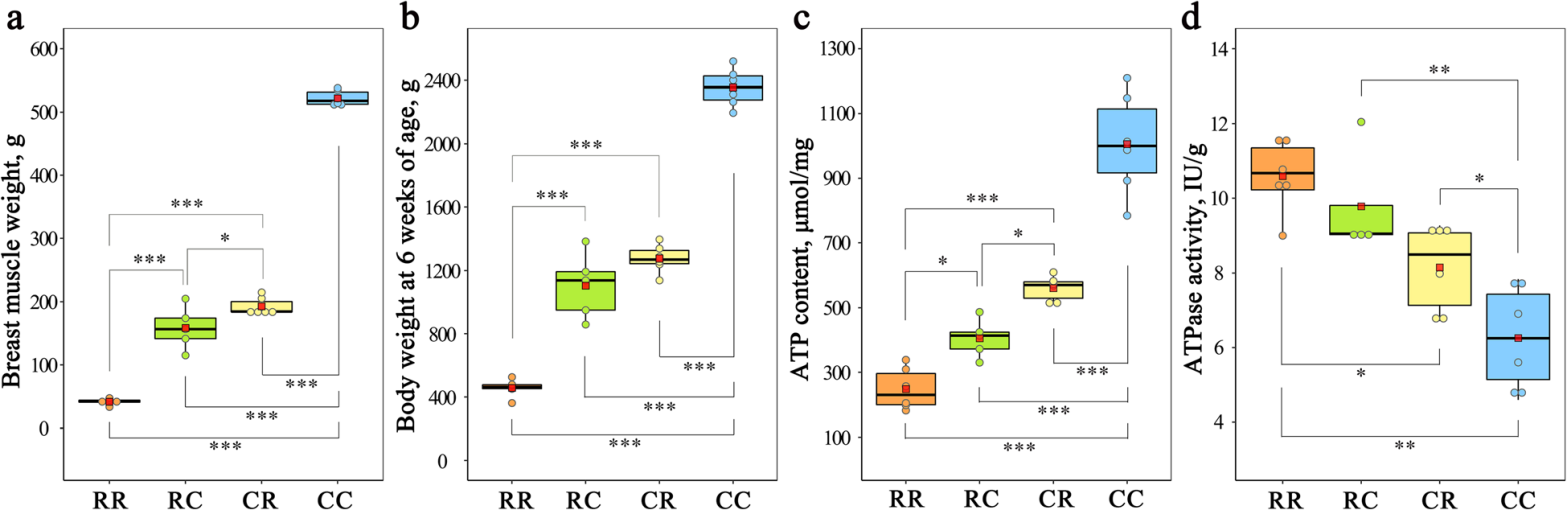
ATP content and ATPase activity detection. Difference analysis of breast muscle weight (**a**), body weight (**b**), ATP content (**c**), and ATPase activity (**d**) among RR, RC, CR, and CC. For **a-d**, each dot represents a sample. The central red dot represents the mean value of the corresponding group. ***, **and * indicate adjusted *P*-values less than 0.001, 0.01, and 0.05, respectively

The gene expression pattern results showed that nonadditive genes were related to the biological process of oxidative phosphorylation, implying that energy metabolism plays a vital role in negative heterosis of breast muscle. To further confirm the relationship between oxidative phosphorylation and negative heterosis for growth traits, we detected the ATP concentration and hydrolysis activity of breast muscle tissues for the CC, RR, CR, and RC groups. ATPases are a group of enzymes that catalyze the hydrolysis of ATP to form ADP. The detection of ATP content and ATPase activity revealed that the group with lower growth traits had lower ATP content but higher ATPase activity (Fig. 6a-d). As shown in Fig. 6a, breast muscle mass was significantly higher in CC than in RR, RC, and CR. The same trend was observed in body weight (Fig. 6b) and ATP content (Fig. 6c), and an opposite trend for ATPase activity is presented in Fig. 6d.

## Discussion

The utilization of heterosis has contributed tremendously to the increased productivity in many domesticated animals and crops for decades. In terms of the calculation formula, H% can be a positive or negative sign. Compared with the extensive studies on positive heterosis [11, 13, 43–45], the phenomenon of negative heterosis is overlooked in breeding programs and genetic studies, even though it exists widely in nature. In the present study, we observed that negative heterosis of body weight and carcass traits in juvenile chickens was more common than positive heterosis. This phenomenon was also reported by Williams et al. [25], Liu et al. [28] and Sutherland et al. [46]. Among the carcass characteristics, we found that meat production displayed the largest negative heterosis in reciprocal crosses of females and males. The H% of breast and drumstick muscle weight was over − 40% and − 20%, respectively. These results were consistent with previous research showing that the negative heterosis of breast muscle weight in crosses was the largest among carcass traits when using broilers and layers as parents [30]. Positive or negative heterosis does not imply superiority or inferiority since it depends on the trait’s biological significance and production preference [25, 34]. In livestock production, the negative heterosis of growth and meat yield was unfavorable since it reduced the edible carcass portions. We characterized the transcriptome profiles of breast muscle in reciprocal crosses and the parental lines herein to reveal the potential mechanisms of negative heterosis for growth traits in chickens.

A large number of DEGs between reciprocal crosses and the parental lines were identified. The number of DEGs between two parental lines was greater than that between reciprocal crosses and their parental lines. This result was consistent with a previous study [11] and indicated that the genetic difference between two parental lines was larger than that between reciprocal crosses and their parental lines. Nonadditive genetic variance can result from a nonlinear phenotypic effect of alleles at one locus, as in the case of dominant or recessive allele pairs in classical genetics. Thus, the nonadditive expression pattern is critically important to the formation of heterosis [2, 14]. Recently, gene expression pattern analysis of chicken liver tissues revealed that overdominant genes related to lipid metabolism played a central role in the heterosis of fat deposition [34]. Wu et al. [33] reported that dominant genes involved in carbohydrate metabolism were associated with heterosis for body weight in *Drosophila melanogaster*. In the present study, we classified these DEGs between reciprocal crosses and their parental lines into additivity, dominance and overdominance. Our results revealed that nonadditivity, including dominance and overdominance, was the major gene expression pattern in reciprocal crosses. Similar results were observed in *Arabidopsis* [2]*, Crassostrea gigas* [14] and chickens [34]*.* Previous reports in *Medicago sativa* [47] and *Larix kaempferi* [48] showed that the proportion of nonadditive genes in heterotic hybrids was higher than that in nonheterotic hybrids. It should be noted that nonadditive genes accounted for 76% of DEGs in the RC group, which was more than that observed in the CR group (69%), and the degree of negative heterosis for growth traits in the RC group was higher than that in the CR group. These results implied that the magnitude of the heterotic response was related to the proportion of genes with nonadditive expression.

To better understand the molecular basis of negative heterosis, functional enrichment analysis was performed to gain insight into the biological relevance of nonadditive inheritance in reciprocal crosses. We found that the process of oxidative phosphorylation was significantly enriched in nonadditive genes of reciprocal crosses, indicating the special and crucial roles of energy metabolism in the negative heterosis of growth traits. Several previous studies have described the correlation of oxidative phosphorylation with heterosis in corn [49], wheat [50] and rice [12]. Seymour et al. [2] found that the growth-related traits of *Arabidopsis* hybrids were associated with energy production via oxidative phosphorylation. This association was also reported in animals. McDaniel and Grimwood [51] demonstrated that heterosis of body weight in *Drosophila melanogaster* was correlated with oxidative phosphorylation efficiency. To validate the role of oxidative phosphorylation in negative heterosis of muscle yield in chickens, we further detected the ATP content and ATPase activity of breast muscle tissues for reciprocal crosses and parental lines. ATPase, as an essential enzyme in energy metabolism, catalyzes the hydrolysis of ATP to form ADP and harnesses the energy released from the breakdown of the phosphate bond to perform other cellular reactions. Our results showed that chickens with lower breast muscle weight had lower ATP content but higher ATPase activity, suggesting that chickens with higher ATP consumption had lower meat production. This finding corroborated that energy metabolism contributed strongly to negative heterosis and might help provide effective strategies for reducing the rate of ATP hydrolysis to improve muscle yield in crossbreds. Since the expression level of nonadditive genes involved in oxidative phosphorylation of reciprocal progenies biased to the R line (egg-type chicken), the expression level in the R line was significantly higher than that in the C line. Thus, the objective of reducing the rate of ATP hydrolysis might be achieved by decreasing the difference in parental weights or increasing the proportion of broiler parentage in the crossbred population.

Growth is a complex polygenetic trait. To identify significant genes underlying the observed negative heterosis, we extracted the nonadditive genes detected in the process of oxidative phosphorylation in reciprocal crosses. A total of 31 shared genes were detected in reciprocal crosses. These genes encoding NADH dehydrogenase, cytochrome c reductase, cytochrome c oxidase, ATP synthase, ATPase and succinate dehydrogenase were all reported to be involved in the regulation of muscle growth and development, such as ATP5C1 [52], ATP5G3 [53], ATP5H [54, 55], ATP5J [53], ATP6AP1 [56], COX6B1 [57], NDUFA1 [52], NDUFA4 [52], NDUFA5 [52], NDUFA6 [52], NDUFV2 [53, 58], NDUFS6 [53], UQCR10 [52], UQCR11 [52], UQCRFS1 [57], SDHA [52, 53, 57] and SDHB [52, 53, 58]. Among these shared genes in reciprocal crosses, more than 60% of nonadditive genes exhibited a similar expression pattern to the layer line. The growth rate and body weight of layer chickens are considerably lower than those of broilers. It might be the large disparity of growth between layers and broilers and the differences in resource allocations that led to negative heterosis of growth traits in crossbred progenies. The Galgal5 may not be optimal chicken genome reference due to GRCg6a is available now, but should be sufficient to draw a conclusion that the important role of nonadditive genes and their related oxidative phosphorylation in negative heterosis for growth traits in chickens, since we confirmed the results by the detection of ATP content and ATPase activity. However, Gene expression is a dynamic process [59], and our research focused on gene expression analysis in juvenile chickens. We expect to determine whether the contributions of nonadditive genes would persist over time and to what degree they would impact the heterosis of growth traits in future experiments. In addition, the negative heterosis of growth traits in males was similar to that observed in females. However, sex-linked factors [60, 61], such as hormones, may influence the growth rate. Therefore, further experiments should be performed to confirm that nonadditive genes and their related oxidative phosphorylation are also the major genetic and molecular factors in the negative heterosis of growth in males.

## Conclusions

Our research focused on the phenomenon of heterosis in chickens and found that negative heterosis of growth traits was more common than positive heterosis, especially for muscle yield. Whole genome-wide gene expression pattern analysis showed that nonadditivity was the major mode of gene action in crossbred chickens. Nonadditive genes related to the biological process of oxidative phosphorylation played a critical role in the formation of negative heterosis for growth traits. Chickens with higher ATP consumption had lower muscle production. Our study revealed fundamental mechanisms of negative heterosis for growth traits in chickens and has important implications for muscle yield improvement.

## Supplementary Information

**Additional file 1: Figure S1.** Correlation among body weights of females and males at different ages. **Figure S2.** Correlation among body weight and muscle mass at 6 weeks of age. **Figure S3.** Volcano plot of differentially expressed genes between reciprocal crosses and parental lines. **Figure S4.** KEGG pathway analysis of overdominant genes in reciprocal crosses.

**Additional file 2: Table S1.** Descriptive statistics for body weight of females and males from hatch to 8 weeks of age. **Table S2.** Descriptive statistics for the left shank and sternum length of females and males during the experiment. **Table S3.** Summary statistics for transcriptome sequencing data. **Table S4.** Classification of different expression patterns of genes. **Table S5.** Descriptive statistics for heterosis of the length of shank and sternum for females and males during the experiment. **Table S6.** Heterosis of carcass performance for F_1_ progenies at 6 weeks of age. **Table S7.** Gene expression patterns of differentially expressed genes in reciprocal crosses. **Table S8.** GO terms of the top 20 overdominant genes in the RC and CR groups. **Table S9.** Detailed information on the overlap of nonadditive genes in RC and CR groups.

## Data Availability

The RNA sequencing data are available from the Sequence Read Archive (https://www.ncbi.nlm.nih.gov/sra) with BioProject number PRJNA524721.

## Abbreviations

C: Cornish
CC: The purebred progenies that used Cornish as parents
CR: The crossbred progenies that used Cornish as the paternal line and Rhode Island White as the maternal line
DEGs: Differentially expressed genes
FPKM: Fragments per kilobase million
GO: Gene Ontology
H%: Heterosis as a percentage
KEGG: Kyoto Encyclopedia of Genes and Genomes
R: Rhode Island White
RC: The crossbred progenies that used Rhode Island White as the paternal line and Cornish as the maternal line
RR: The purebred progenies that used Cornish as parents
PCA: Principal component analysis
qRT-PCR: Quantitative real-time PCR
