# Supplementary material for: Genetic basis of negative heterosis for growth traits in chickens revealed by genome-wide gene expression pattern analysis
Source: J Anim Sci Biotechnol. 2021 Apr 18;12:52. doi: 10.1186/s40104-021-00574-2 (PMC8053289; doi:10.1186/s40104-021-00574-2)
Supplement: Supplementary file 1 — Additional file 1: Figure S1. Correlation among body weights of females and males at different ages. Figure S2. Correlation among body weight and muscle mass at 6 weeks of age. Figure S3. Volcano plot of differentially expressed genes between reciprocal crosses and parental lines. Figure S4. KEGG pathway analysis of overdominant genes in reciprocal crosses. [file 40104_2021_574_MOESM1_ESM.docx]

# Supplementary Figures

**
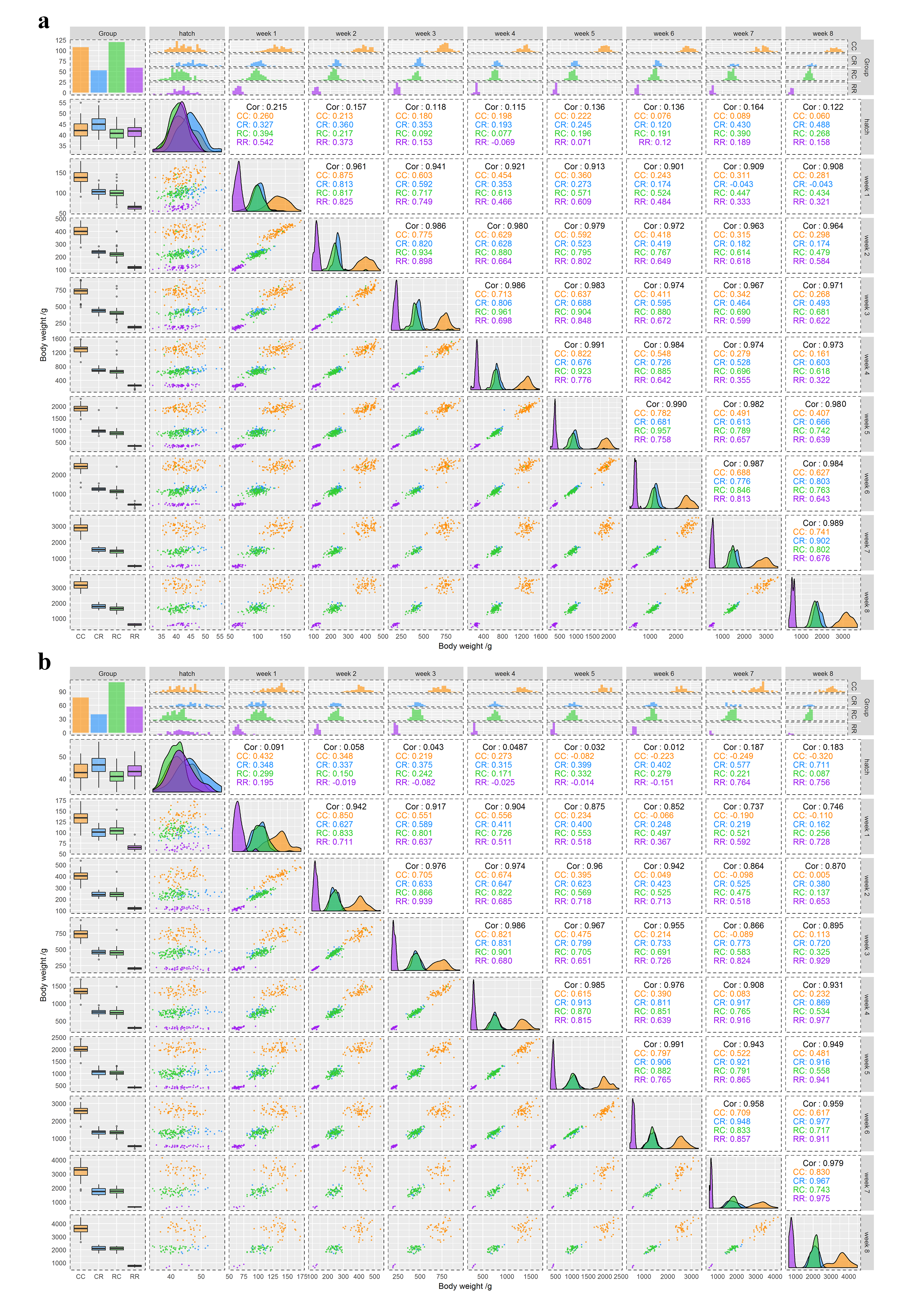
**

**Figure S1. Correlation among body weights of females and males at different ages. (a)** Females; **(b)** Males. The sample number of the four groups (CC, CR, RC, and RR) and the distribution of body weight from hatch to 8 weeks of age are shown on the upper side and in the diagonal line. The correlation coefficient among body weight at different ages is displayed on the upper panel above the diagonal. The scatter plots of body weight for four groups from hatch to 8 weeks of age are shown on the lower panel below the diagonal. Box plots of body weight for four groups from hatch to 8 weeks of age are displayed on the left side.


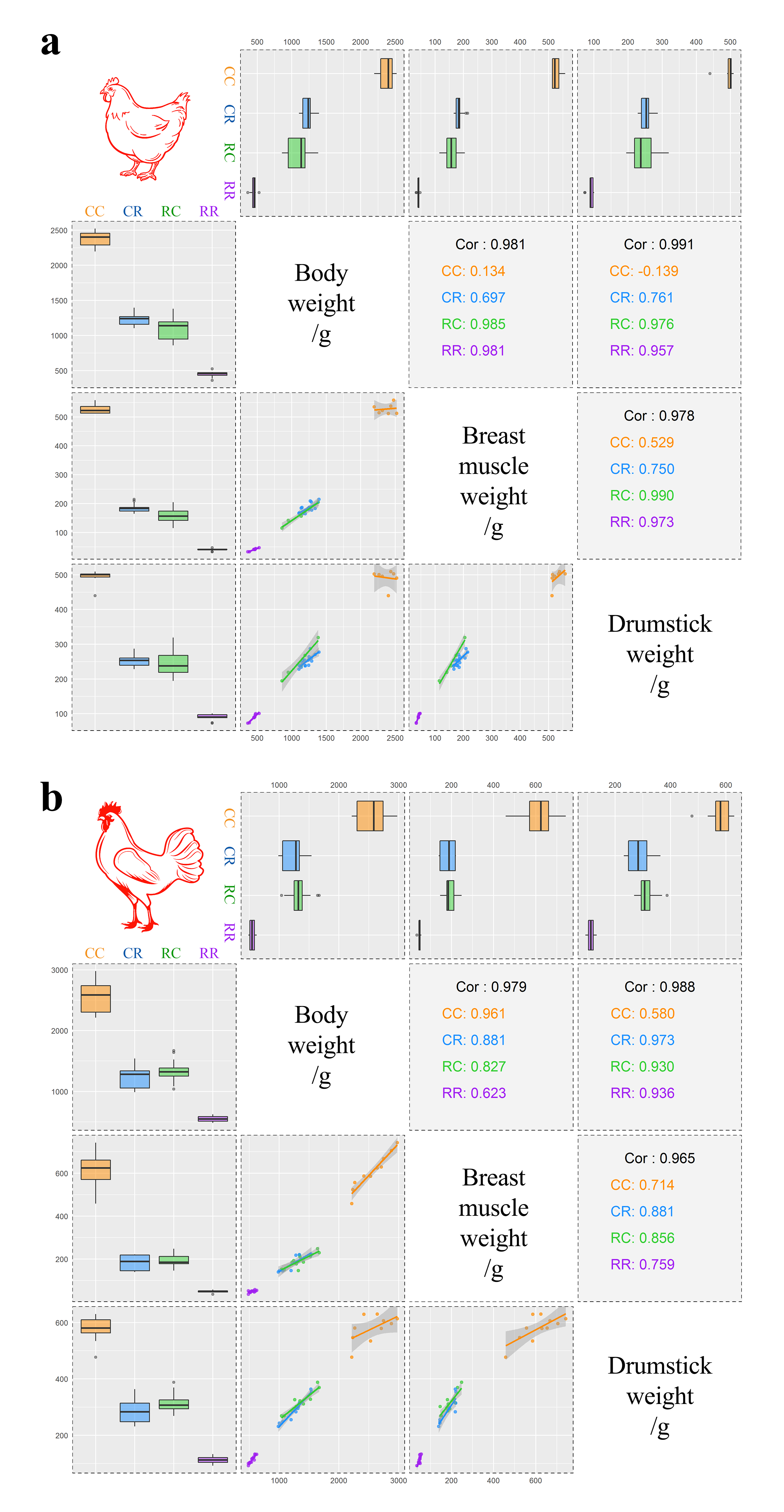


**Figure S2. Correlation among body weight and muscle mass at 6 weeks of age. (a)** Females; **(b)** Males. The box plots of body weight for four groups at 6 weeks of age are displayed on the left and upper sides. The correlation coefficient of body weight and muscle mass at 6 weeks was displayed on the upper panel above the diagonal. The scatter plots of body weight and muscle mass at 6 weeks are shown on the lower panel below the diagonal.

**
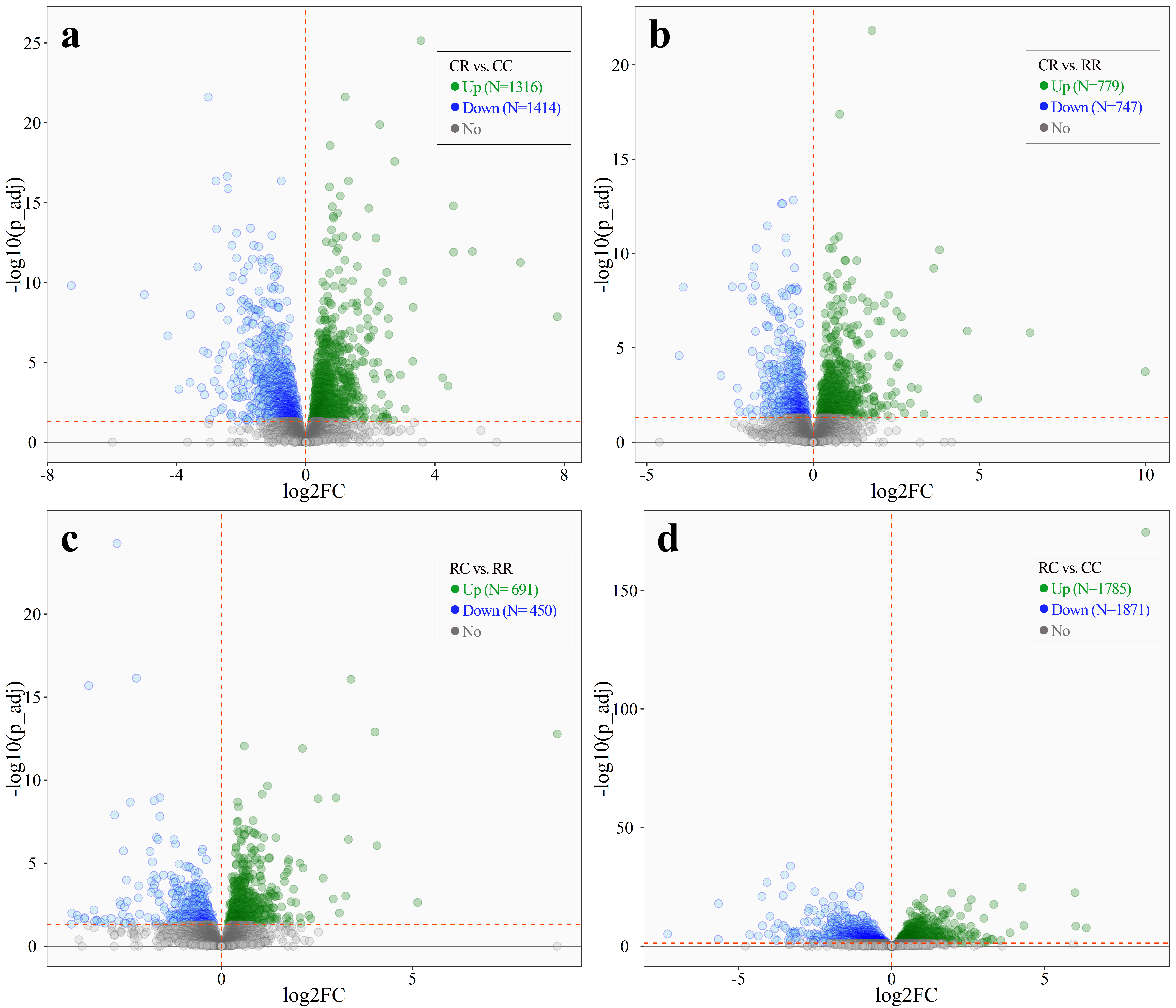
**

**Figure S3. Volcano plot of differentially expressed genes between reciprocal crosses and parental lines. (a)** between CR and CC; **(b)** between CR and RR; **(c)** between RC and RR; **(d)** between RC and CC.

**
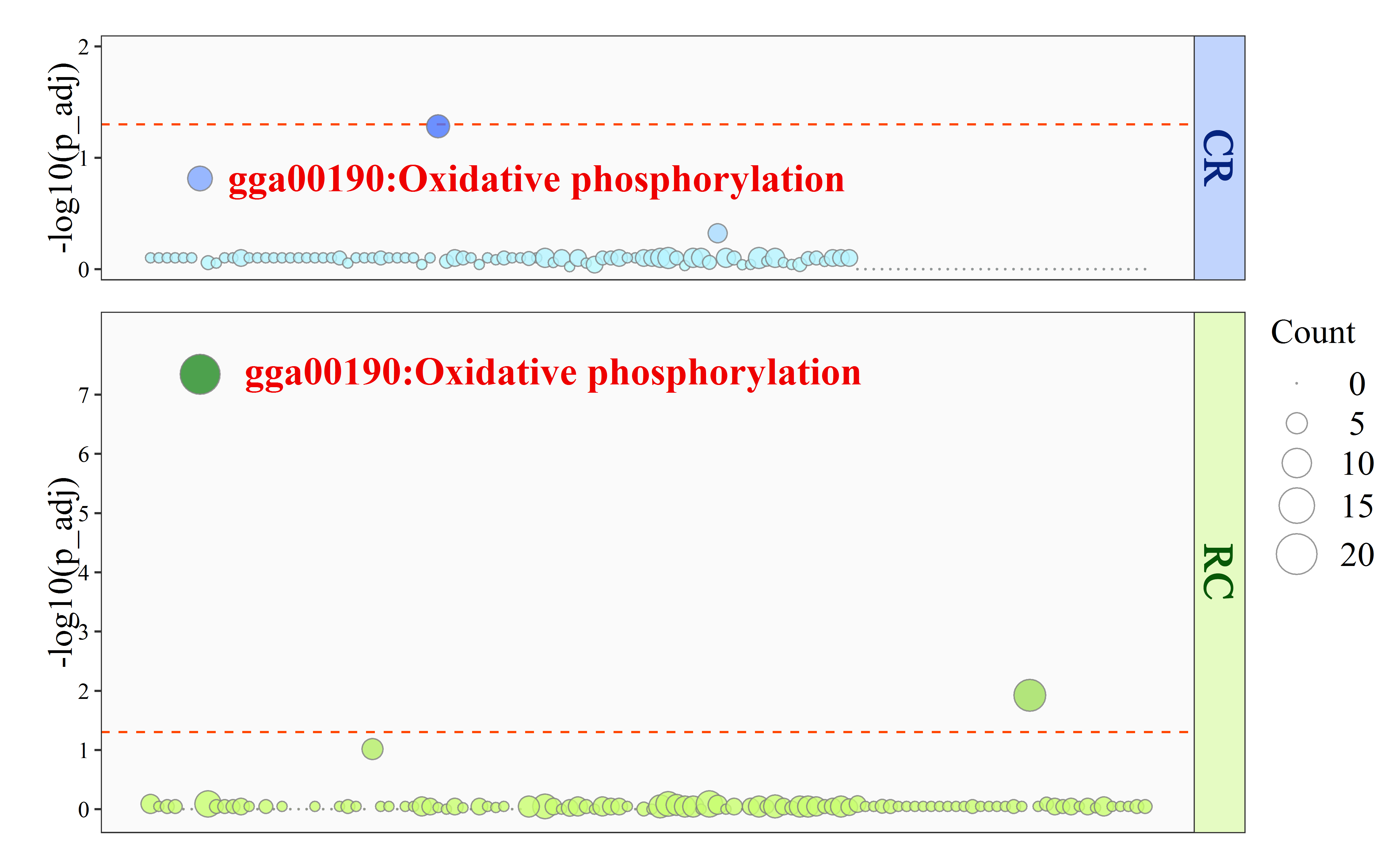
**

**Figure S4. KEGG pathway analysis of overdominant genes in reciprocal crosses.**
